# Activation of the NF-*κ*B and MAPK Signaling Pathways Contributes to the Inflammatory Responses, but Not Cell Injury, in IPEC-1 Cells Challenged with Hydrogen Peroxide

**DOI:** 10.1155/2020/5803639

**Published:** 2020-01-21

**Authors:** Kan Xiao, Congcong Liu, Zhixiao Tu, Qiao Xu, Shaokui Chen, Yang Zhang, Xiuying Wang, Jing Zhang, Chien-An Andy Hu, Yulan Liu

**Affiliations:** ^1^Hubei Key Laboratory of Animal Nutrition and Feed Science, Hubei Collaborative Innovation Center for Animal Nutrition and Feed Safety, Wuhan Polytechnic University, Wuhan, China; ^2^Department of Biochemistry and Molecular Biology, University of New Mexico School of Medicine, Albuquerque, NM 87131, USA

## Abstract

Oxidative stress can lead to intestinal cell injury as well as the induction of inflammation. It is not clear whether inflammation is an important factor leading to cell injury caused by oxidative stress. The purpose of this study was to investigate the role of inflammation in intestinal injury caused by hydrogen peroxide (H_2_O_2_). Our results revealed that H_2_O_2_ stimulation significantly decreased the viability of intestinal porcine epithelial cells (IPEC-1), increased lactate dehydrogenase (LDH) activity, and disrupted the distribution of the tight junction protein claudin-1. H_2_O_2_ significantly increased the mRNA expression of interleukin-6 (IL-6), IL-8, and tumor necrosis factor-*α* (TNF-*α*). H_2_O_2_ stimulation also led to increased phosphorylation of p38 and jun N-terminal kinase (JNK), and p65 NF-*κ*B protein translocation into the nucleus of IPEC-1 cells. Cells treated with the NF-*κ*B inhibitor (BAY11-7082), the p38 inhibitor (SB202190), or the JNK inhibitor (PD98059) significantly decreased mRNA and protein expression of IL-6, IL-8, and TNF-*α*. However, treatment with mitogen-activated protein kinase (MAPK) or NF-*κ*B inhibitors did not prevent the damage effect on cell viability, LDH activity, or the distribution of claudin-1 in cells challenged with H_2_O_2_. In summary, our data demonstrate that activation of the NF-*κ*B and MAPK signaling pathways can contribute to the inflammatory response, but not cell injury, in IPEC-1 cells challenged with H_2_O_2_.

## 1. Introduction

The intestinal barrier is the first line of defense inside the body against harmful antigens and pathogens, within the intestinal lumen [1, 2]. It is a single layer of cells lining the gut, consisting of enterocyte membranes and tight junctions between enterocytes. The integrity of the intestinal barrier is essential for the digestion and absorption of nutrients, both in humans and animals [3–5]. Intestinal epithelial cells establish a barrier between the hostile external environment and the internal milieu. However, numerous factors such as infection, inflammation, and oxidative stress can result in intestinal epithelia injury, and dysfunction [1].

Hydrogen peroxide (H_2_O_2_) is a highly reactive oxygen specie and serves as a signaling molecule in the regulation of a wide variety of biological processes [6–8]. Excessive H_2_O_2_ is capable of diffusing throughout mitochondria and across cell membranes, where it can cause multiple forms of cellular oxidative injury [9, 10]. Oxidative stress can lead to intestinal cell injury by various means [11, 12]. It can cause cell damage by free radicals or by induction of inflammation [13]. Oxidative stress appears to be an important driving force for an enhanced cytokine production in intestinal epithelial cells, resulting in gut mucosal inflammation [14]. However, it is not clear whether inflammation is an important factor leading to cell injury caused by oxidative stress. Mitogen-activated protein kinase (MAPK) and NF-*κ*B pathways are common inflammatory signaling pathways. Activation of MAPKs and NF-*κ*B pathways, caused proinflammatory cytokines such as interleukin-6 (IL-6), IL-8, and tumor necrosis factor-*α* (TNF-*α*) to be released, which could lead to the inflammatory response [15]. Traditionally, it is thought that cell death is the most extreme form of injury produced by inflammation [16]. Inflammation and cell death are distinct but highly interconnected and mutually regulated host-mediated mechanisms, which are important for maintaining homeostasis in organisms [17].

Therefore, the aim of the present study was to investigate the role of inflammation in intestinal injury caused by H_2_O_2_. It was found that H_2_O_2_ induced cell injury, upregulated proinflammatory cytokine-related gene expression, and activated NF-*κ*B, and MAPK signaling pathways in intestinal porcine epithelial cells (IPEC-1). In addition, the inhibition of p38, JNK, and NF-*κ*B with selective inhibitors downregulated the inflammatory response but did not prevent cell injury in IPEC-1 cells. Our data demonstrated that activation of the NF-*κ*B and MAPK signaling pathways could contribute to the inflammatory responses but not cell injury in IPEC-1 cells challenged with H_2_O_2_.

## 2. Materials and Methods

### 2.1. Cell Reagents and Antibodies

IPEC-1 cells were derived from the jejunum/ileum of a neonatal unsuckled piglet and were kindly provided by Dr. Guoyao Wu's laboratory at Texas A&M University. These cells were used for all experiments and were cultured in Dulbecco's Modified Eagle's Medium-F12 (HyClone, St. Louis, MO, USA), supplemented with 5% fetal bovine serum (Sigma-Aldrich, St. Louis, MO, USA), 1% insulin-transferrin-selenium, 1% penicillin/streptomycin (Gibco, NY, USA), and epidermal growth factor (5 ng/mL) (Gibco, NY, USA). Cells were split into 75 cm^2^ culture flasks when cell monolayers reached 70%-80% confluency using trypsin/EDTA (0.25%, 0.9 mM EDTA) (Gibco, NY, USA). The cells were incubated at 37°C in a humidified atmosphere of 5% CO_2_, and culture medium was changed every second day, according to standard culture protocols. The MAPK inhibitors (p38 inhibitor SB202190 and JNK inhibitor PD98059) and NF-*κ*B inhibitor (Bay11-7082) were purchased from Sigma-Aldrich (St. Louis, MO, USA). The MAPK antibodies and anti-phospho-MAPK antibodies were purchased from Cell Signaling Technology, Inc. (Danvers, MA, USA). The secondary antibody was horseradish peroxidase-conjugated anti-rabbit IgG (Santa Cruz Biotechnology, Santa Cruz, CA, USA).

### 2.2. Cell Viability Assays

Cell viability was detected using the Cell Counting Kit-8 detection kit (CCK-8, Beyotime Institute of Biotechnology, Wuhan, CH). Cells were cultured in 96-well plates. When they were cultured to 70%-80% confluency, medium was removed and after washing two times with phosphate-buffered saline (PBS), cells were treated with different concentrations of H_2_O_2_ (0, 0.2, and 0.5 mM) for 3 h, or pretreated with different inhibitors (Bay11-7082, SB202190, and PD98059) for 1 h, in the presence or absence of 0.5 mM H_2_O_2_ for 3 h. Dulbecco's Modified Eagle's Medium-F12 medium was added to the control group. After a 3 h incubation, media was removed and 10% CCK-8 reagent (100 *μ*L per well) was added and incubated at 37°C for 1 h according to the manufacturer's instructions. The absorbance was quickly measured at a wavelength of 450 nm using a microplate reader (Bio-Rad, CA, USA). The viability of the cells was recorded as the percentage relative to untreated controls. All assays were performed in triplicate and in at least three independent experiments.

### 2.3. Lactate Dehydrogenase (LDH) Activity Measurement

The concentration of LDH released into the culture medium through damaged cell membranes was measured spectrophotometrically using an LDH assay kit (Nanjing Jiancheng Bioengineering Institute, Nanjing, China). IPEC-1 cells were cultured in 12-well plates until 80% confluent. Cells were then exposed to H_2_O_2_ (0, 0.2, and 0.5 mM) or pretreated with different inhibitors (Bay11-7082, SB202190, or PD98059) for 1 h, in the presence or absence of 0.5 mM H_2_O_2_ for 3 h. Subsequently, cell supernatants were collected for LDH measurement according to the manufacturer's protocol. The absorbance was read at a wavelength of 450 nm using an automated microplate reader (Bio-Rad, CA, USA).

### 2.4. Quantitative Real-Time PCR

To investigate the effects of H_2_O_2_ or the inhibitors on proinflammatory cytokine expression, we used 0.5 mM H_2_O_2_ to treat the cells or cells were pretreated with different inhibitors (Bay11-7082, SB202190, or PD98059) for 1 h, in the presence or absence of 0.5 mM H_2_O_2_ for 3 h; cells were then collected for mRNA expression analysis. Messenger RNA levels were determined by real-time PCR as described by [16]. After washing IPEC-1 cells two times with ice-cold PBS, total RNA was extracted using the RNAiso Plus Kit (TaKaRa Biotechnology (Dalian) Co., Ltd., Dalian, China) following the manufacturer's instructions. After purification and quantitation, reverse transcription was performed using the PrimeScript® RT Reagent Kit (TaKaRa Biotechnology (Dalian) Co., Ltd., Dalian, China) following the manufacturer's instructions. Quantitative analysis of PCR was carried out on a ABI 7500 Real-Time PCR System (Applied Biosystems, Life Technologies) using a SYBR® Premix Ex Taq™ qPCR Kit (TaKaRa Biotechnology (Dalian) Co., Ltd., Dalian, China). The primer pairs used are listed in Table 1. Results were analyzed by the 2^−ΔΔCT^ method with GAPDH as the housekeeping gene as GAPDH displayed no variation among all groups [16]. All samples were run in triplicate. Relative mRNA abundance of each target gene was normalized to the control group.

### 2.5. Cytokine Production Measurement

IPEC-1 cells were seeded in 12-well plates and treated with different concentrations of H_2_O_2_ (0 and 0.5 mM) for 3 h or pretreated with different inhibitors (Bay11-7082, SB202190, and PD98059) for 1 h, in the presence or absence of 0.5 mM H_2_O_2_ for 3 h. Subsequently, cell supernatants were collected for cytokine IL-6, IL-8, and TNF-*α* measurement by ELISA using commercial kits (4A Biotech, Bejing, China) according to the manufacturer's protocol.

### 2.6. Western Blot

To evaluate the effects of H_2_O_2_ on MAPK signaling activation, we used the 0.5 mM concentrations of H_2_O_2_ to treat IPEC-1 cells. After incubation for 3 h, we collected the cells for Western blot analysis. Cells were then lysed and centrifuged, and the supernatants were collected for western blot and protein assay according to our previous study [16]. After transmembrane electrophoresis, blots were incubated with primary antibodies against ERK, p38, JNK, phospho-ERK (p-ERK), phospho-p38 (p-p38), and phospho-JNK1/2 (p-JNK1/2) (Cell Signaling Technology, Inc., Danvers, MA, USA) overnight at 4°C and then incubated with a secondary antibody (Santa Cruz Biotechnology, Santa Cruz, CA, USA) at 25°C for 2 h. The blots were visualized with an enhanced Chemiluminescence Western Blot Kit (Amersham Biosciences) and processed with the Quantity One® software (Bio-Rad, CA, USA). The results were expressed as the abundance of p-ERK, p-p38, and p-JNK1/2 relative to the total protein content of ERK, p38, and JNK, respectively.

### 2.7. Immunofluorescence

IPEC-1 cells were seeded onto 12-well glass coverslips (Corning, MA, USA). Cells were then exposed to H_2_O_2_ (0 or 0.5 mM) for 3 h when the cells reached 70%-80% confluency. After fixing and blocking, the cells were then incubated with primary rabbit anti-NF-*κ*B p65 antibody overnight at 4°C, followed by incubation with the secondary Cy3-labelled antibody in a humidified chamber for 1 h at room temperature. Subsequently, the cells were stained with 2-(4-amidinophenyl)-6-indolecarbamidine dihydrochloride solution. Each of the steps above was followed by washing three times for 5 mins each in cold PBS. Finally, the IPEC-1 cells on coverslips were preserved in antifade mounting medium. The activation and nuclear translocation of NF-*κ*B were observed using a fluorescent microscope (Olympus, Tokyo, Japan).

### 2.8. Confocal Laser Scanning Microscope Analysis

IPEC-1 cells were seeded onto glass microscope coverslips (Corning, MA, USA). To assess the effect of inhibitors on H_2_O_2_-induced barrier function, cells were pretreated with SB202190, PD98059, or Bay11-7082 for 1 h and then challenged with H_2_O_2_ when cells reached 70%-80% confluency. All cells were fixed with 4% paraformaldehyde for 10 min at room temperature (RT) and permeabilized with 0.2% Triton X-100 in PBS at RT for 10 min. The cells were blocked with 1% bovine serum albumin in PBS for 30 min at RT and then incubated with anti-claudin-1 antibody overnight. After being washed five times in PBS, the cells were incubated for 2 h at RT with a goat anti-rabbit secondary antibody conjugated to Alexa Fluor 488 (Invitrogen, CA, USA), followed by counterstaining with 4,6-diamidino-2-phenylindole (Sigma-Aldrich, St. Louis, MO, USA). The coverslips were mounted onto glass microscope slides using mounting buffer, and the stained cells were visualized using a fluorescent confocal laser scanning microscope (Carl Zeiss, Gottingen, Germany).

### 2.9. Statistical Analysis

Data were analyzed by Student's *t*-test or ANOVA (IBM Institute, Chicago, IL, USA). For the inhibitor trial, the effects of inhibitors and H_2_O_2_ were analyzed as a 2 × 2 factorial arrangement using the general linear model (GLM) procedures. All data were presented as means with standard errors of means. The statistical model included the effects of inhibitors, H_2_O_2_, and their interactions. When significant inhibitor × H_2_O_2_ interaction or a trend for inhibitor × H_2_O_2_ interaction occurred, multiple comparison tests were performed using ANOVA. All data were represented by at least three independent experiments. Differences were considered significant for values of *P* ≤ 0.05.

## 3. Results

### 3.1. H_2_O_2_ Induces Cell Injury in IPEC-1 Cells

#### 3.1.1. Cell Viability

**Figure 1:**
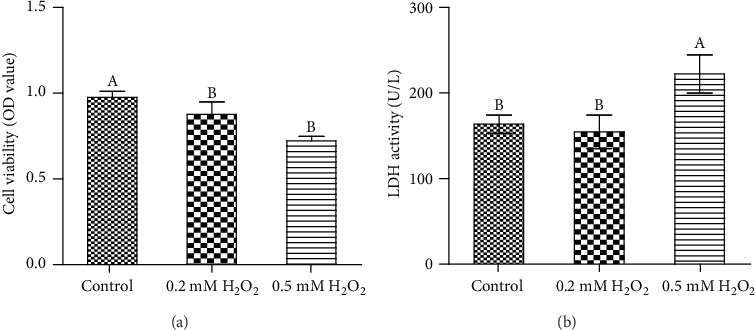
Effects of H_2_O_2_ on cell viability and lactate dehydrogenase activity in IPEC-1 cells. (a) Cell viability. (b) LDH activity. ^AB^Means within a figure with different letters indicate H_2_O_2_ treatment and the control group differs significantly (*P* ≤ 0.05).

To explore the effect of H_2_O_2_ on cell injury, we used the CCK-8 kit to determine cell viability after treatment with 0, 0.2 mM, and 0.5 mM H_2_O_2_ for 3 h. The dose and time point used were determined based on our preliminary experiments (data not shown). Our results demonstrated that 0.2 mM and 0.5 mM H_2_O_2_ stimulation significantly decreased cell viability in IPEC-1 cells (Figure 1(a)).

#### 3.1.2. LDH

This enzyme is found in virtually all living cells, is released extracellularly when cells are damaged, and is commonly used as a marker of cell injury. In our study, compared with control cells, 0.5 mM H_2_O_2_ stimulation significantly increased the LDH activity in the cell supernatant (Figure 1(b)). This data, in combination with a cell viability indicator, led us to use 0.5 mM of H_2_O_2_ in all subsequent experiments.

#### 3.1.3. Tight Junction Protein

**Figure 2:**
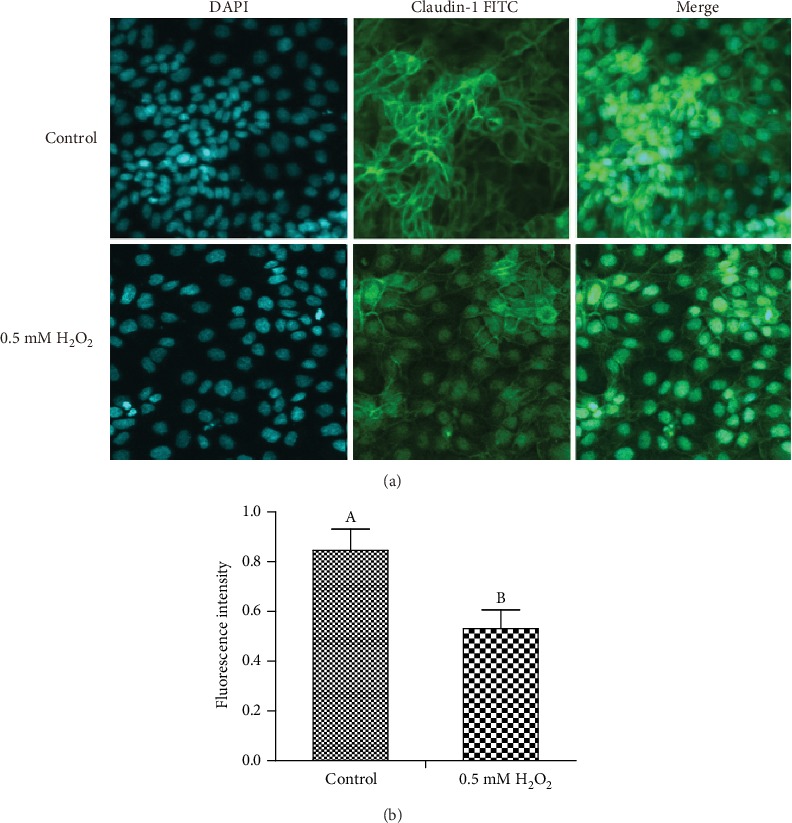
Effect of H_2_O_2_ on tight junction protein claudin-1 distribution in IPEC-1 cells. (a) Representative picture of claudin-1 protein distribution. (b) Quantification of claudin-1. DAPI: 4′,6-diamidino-2-phenylindole (blue); FITC: fluorescein isothiocyanate (green). The cells were examined using a confocal laser microscope at 60x magnification. ^AB^Means within a figure with different letters indicate H_2_O_2_ treatment and the control group differs significantly (*P* ≤ 0.05).

To explore the effect of H_2_O_2_ on cell barrier function, we also assessed the distribution of the tight junction protein claudin-1 in IPEC-1 cells, using confocal microscopy. Claudin-1 protein is one of the most important proteins associated with tight junctions. In the control cells, it is normally distributed uniformly across the cell membrane. Under external stimulation, however, it becomes nonuniformly distributed, both inter and extracellularly. Our results demonstrated that 0.5 mM H_2_O_2_ stimulation for 3 h significantly altered the distribution of claudin-1 in IPEC-1 cells (Figures 2(a) and 2(b)), suggesting a role for H_2_O_2_ in the destruction of the cell barrier.

### 3.2. H_2_O_2_ Upregulates mRNA and Protein Expression of Inflammatory-Related Genes in IPEC-1 Cells

To verify the effect of H_2_O_2_ on the inflammatory response, we measured the gene and protein expression levels of the proinflammatory cytokines IL-6, IL-8, and TNF-*α* using RT-PCR. Our results demonstrated that compared with the control cells, 0.5 mM H_2_O_2_ significantly increased the mRNA and protein expression of IL-6, IL-8, and TNF-*α* when compared to control (Tables 2 and 3).

### 3.3. H_2_O_2_ Activates MAPK (p38 and JNK Phosphorylation) and NF-*κ*B p65 Signaling in IPEC-1 Cells

**Figure 3:**
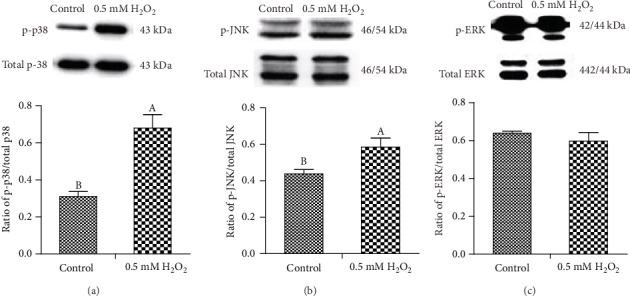
Effect of H_2_O_2_ on p38 (a), JNK (b), and ERK (c) signaling pathway activation (phosphorylation) in IPEC-1 cells. P-MAPK/total MAPK was the ratio reflected by the bars. Values are means (six replicates) with standard errors of means represented by vertical bars. ^AB^Means within a figure with different letters indicate H_2_O_2_ treatment and the control group differs significantly (*P* ≤ 0.05).

**Figure 4:**
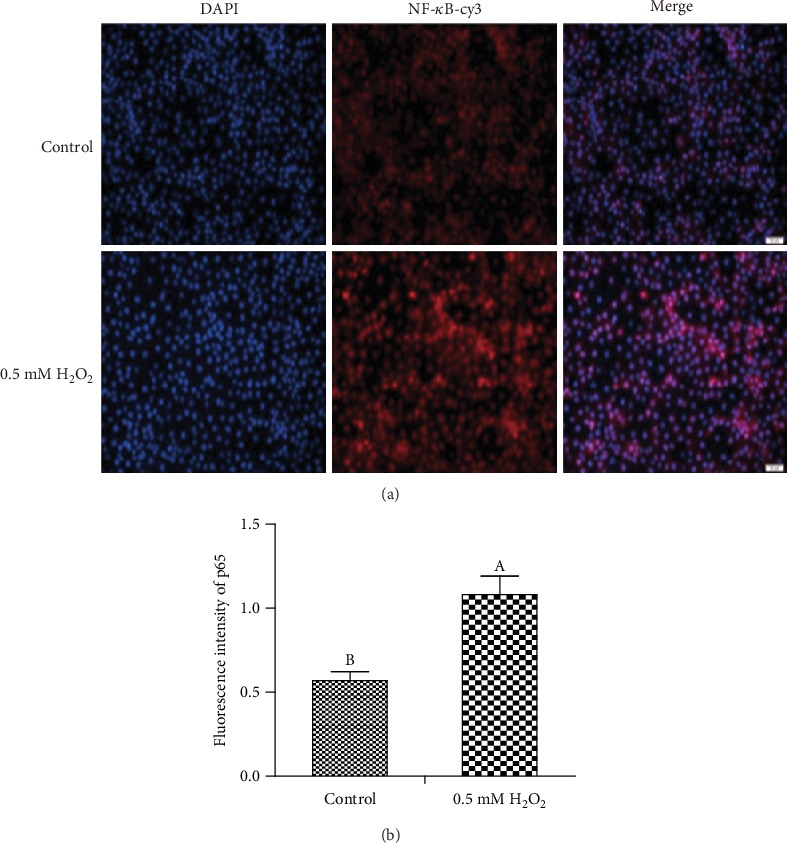
Effect of H_2_O_2_ on NF-*κ*B p65 protein translocation in IPEC-1 cells. NF-*κ*B p65 protein translocation in the IPEC-1 cells challenged with H_2_O_2._ (a) Representative picture of p65 protein translocation (immunofluorescence; ×100). (b) Quantification of p65 translocation. DAPI: 4′,6-diamidino-2-phenylindole; cy3: cyanines. ^AB^Means within a figure with different letters indicate H_2_O_2_ treatment and the control group differs significantly (*P* ≤ 0.05).

To evaluate the activation of MAPK and NF-*κ*B signaling pathways after H_2_O_2_ stimulation, we measured p38, ERK, and JNK phosphorylation and NF-*κ*B p65 protein translocation by western blot analysis and immunofluorescence after incubation with 0.5 mM H_2_O_2_ for 3 h. As demonstrated in Figures 3(a) and 3(b), H_2_O_2_ stimulation significantly increased the phosphorylation of p38 and JNK in IPEC-1 cells compared with control cells. However, H_2_O_2_ challenge had no significant effect on the phosphorylation of ERK and total protein levels for the MAPKs (p38, ERK, and JNK) (Figure 3(c)). Our data also demonstrated that H_2_O_2_ treatment increased NF-*κ*B p65 protein entering the nucleus of IPEC-1 cells (Figures 4(a) and 4(b)). These data demonstrated that H_2_O_2_ activated MAPK (p38 and JNK phosphorylation) and NF-*κ*B p65 signaling pathways in IPEC-1 cells after 3 h incubation.

### 3.4. Inhibition of p38, JNK, and NF-*κ*B with Bay11-7082, SB202190, and PD98059 Downregulates mRNA and Protein Expression of Proinflammatory Cytokine-Related Genes in IPEC-1 Cells

To determine the role of p38, JNK, and NF-*κ*B signaling in inducing the inflammatory response after H_2_O_2_ stimulation, cells were pretreated with specific inhibitors (SB202190, PD98059, or Bay11-7082) or DMSO as control. The time points and concentrations of inhibitors were selected after preliminary time and dose experiments (data not shown). Cells were pretreated with the p38 inhibitor SB202190 (20 *μ*M), JNK inhibitor PD98059 (50 *μ*M), or the NF-*κ*B inhibitor Bay11-7082 (10 *μ*M) for 1 h prior to H_2_O_2_ stimulation. As depicted in Tables 4, 5, and 6, after 3 h incubation, H_2_O_2_ challenge significantly increased the mRNA expression of IL-6, IL-8, and TNF-*α*. However, inhibition of p38, JNK, and NF-*κ*B signaling pathways with Bay11-7082 (Tables 4 and 7), SB202190 (Tables 5 and 8), or PD98059 (Tables 6 and 9) downregulated the mRNA and protein expression of the inflammatory-related genes IL-6, IL-8, and TNF-*α* in IPEC-1 cells. Collectively, the data suggested that the p38, JNK, and NF-*κ*B signaling pathways played an important role in the induction of cell inflammation.

### 3.5. Inhibition of p38, JNK, and NF-*κ*B with Bay11-7082, SB202190, and PD98059 Does Not Prevent Cell Injury in IPEC-1 Cells

#### 3.5.1. Cell Viability

**Figure 5:**
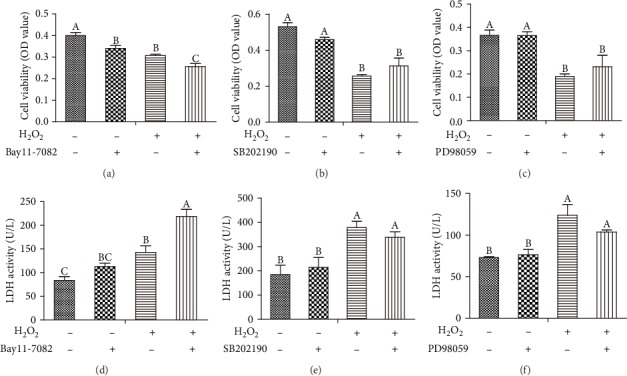
Effects of Bay11-7082, SB202190, and PD98059 on cell viability and LDH activity in the supernatant of IPEC-1 cells stimulated with H_2_O_2_. (a and d) Effects of Bay11-7082 on cell viability and LDH activity in the supernatant of IPEC-1 cells stimulated with H_2_O_2_. (b and e) Effects of SB202190 on cell viability and LDH activity in the supernatant of IPEC-1 cells stimulated with H_2_O_2_. (c and f) Effects of PD98059 on cell viability and LDH activity in the supernatant of IPEC-1 cells stimulated with H_2_O_2_. Values are means with standard errors of means represented by vertical bars. ^ABC^Means within a figure with different letters indicate H_2_O_2_ treatment and the inhibitor group differs significantly (*P* ≤ 0.05).

To further determine a role for p38, JNK, and NF-*κ*B signaling in the induction of the cell injury after H_2_O_2_ stimulation, cells were pretreated with inhibitors (SB202190, PD98059, or Bay11-7082) 1 h prior to a 3 h incubation with H_2_O_2_. Our data demonstrated that H_2_O_2_ stimulation significantly decreased the viability of IPEC-1 cells when compared to control. The inhibitors SB202190, PD98059, and Bay11-7082 did not have a protective effect on cell viability, suggesting that the inhibition of MAPK (p38 and JNK) and NF-*κ*B pathways did not prevent cell injury induced by H_2_O_2_ challenge in IPEC-1 cells (Figures 5(a), 5(b), and 5(c)). Bay11-7082 further decreased the cell viability after H_2_O_2_ stimulation.

#### 3.5.2. LDH

To investigate whether p38, JNK, and NF-*κ*B signaling pathways influenced H_2_O_2_-induced LDH activity in IPEC-1 cells, we pretreated cells with inhibitors (SB202190, PD98059, or Bay11-7082) for 1 h prior to the H_2_O_2_ incubation for 3 h. The results demonstrated that H_2_O_2_ stimulation significantly increased the activity of LDH in the IPEC-1 cell supernatant, and pretreatment with inhibitors (SB202190, PD98059, or Bay11-7082) did not alleviate the increase of LDH activity, indicating that inhibition of the MAPKs (p38 and JNK) and NF-*κ*B signaling pathways did not prevent the H_2_O_2_-induced LDH activity in the IPEC-1 cell supernatant (Figures 5(d), 5(e), and 5(f)). Bay11-7082 further increased LDH activity after H_2_O_2_ stimulation.

#### 3.5.3. Tight Junction Protein

**Figure 6:**
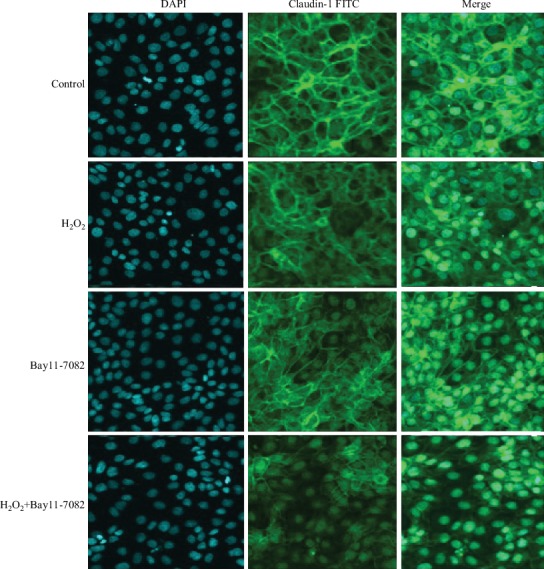
Effect of Bay11-7082 on the protein distribution of claudin-1 in IPEC-1 cells stimulated with H_2_O_2_. DAPI: 4′,6-diamidino-2-phenylindole (blue); FITC: fluorescein isothiocyanate (green). The cells were examined using a confocal laser microscope at 60x magnification.

**Figure 7:**
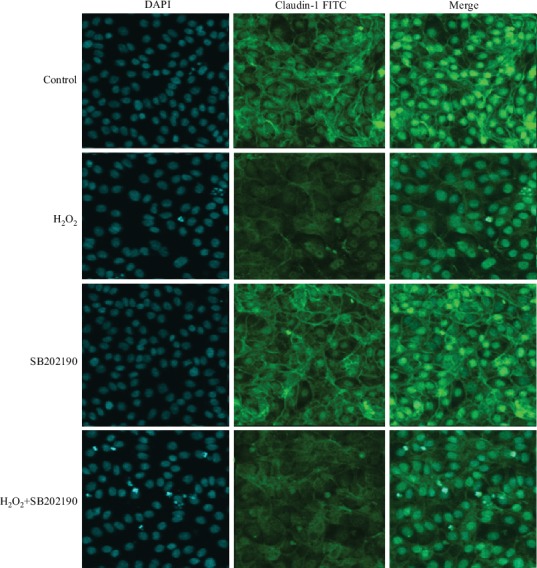
Effect of SB202190 on the protein distribution of claudin-1 in IPEC-1 cells stimulated with H_2_O_2_. DAPI: 4′,6-diamidino-2-phenylindole (blue); FITC: fluorescein isothiocyanate (green). The cells were examined using a confocal laser microscope at 60x magnification.

**Figure 8:**
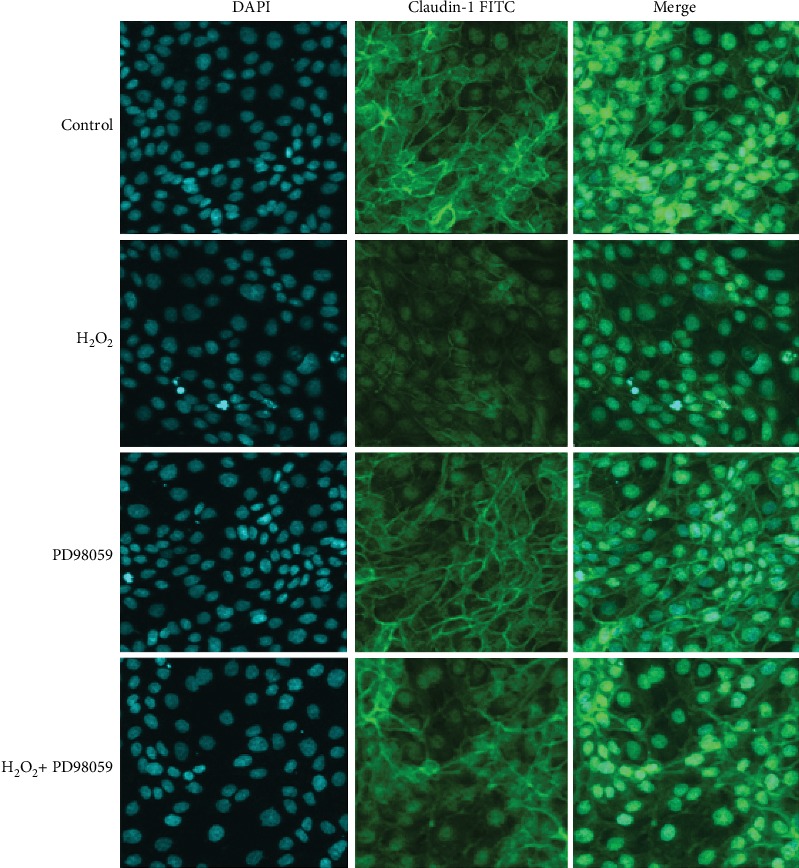
Effect of PD98059 on the protein distribution of claudin-1 in IPEC-1 cells stimulated with H_2_O_2_. DAPI: 4′,6-diamidino-2-phenylindole (blue); FITC: fluorescein isothiocyanate (green). The cells were examined using a confocal laser microscope at 60x magnification.

**Figure 9:**
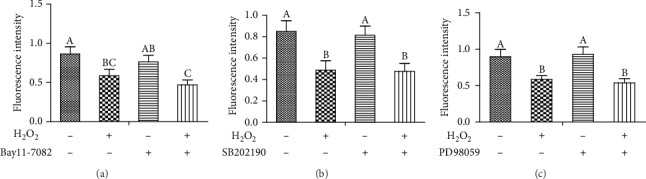
Quantitation of fluorescence intensity of claudin-1 in IPEC-1 cells after NF-*κ*B and MAPK inhibitor treatment. (a) Effect of Bay11-7082 on the protein quantitation of claudin-1 in IPEC-1 cells stimulated with H_2_O_2_. (b) Effect of SB202190 on the protein quantitation of claudin-1 in IPEC-1 cells stimulated with H_2_O_2_. (c) Effect of PD98059 on the protein quantitation of claudin-1 in IPEC-1 cells stimulated with H_2_O_2_. ^ABC^Means within a figure with different letters indicate H_2_O_2_ treatment and the inhibitor group differs significantly (*P* ≤ 0.05).

Next, we sought to identify the role of the p38, JNK, and NF-*κ*B signaling pathways in cell barrier function by using confocal microscopic analysis. As presented in Figures 6, 7, 8, and 9, H_2_O_2_ treatment significantly destroyed the normal distribution of claudin-1 protein in the cells. Pretreating cells with inhibitors (SB202190, PD98059, or Bay11-7082) 1 h prior to the H_2_O_2_ incubation had no effect on the distribution of claudin-1 after H_2_O_2_ challenge. This suggested that inhibition of the MAPKs (p38 and JNK) and NF-*κ*B signaling pathways did not prevent barrier function injury.

## 4. Discussion

The intestinal epithelium performs a dual function as it can absorb dietary nutrients and it can form a physical barrier against noxious stimuli. As the first line of defense, intestinal epithelial cells also participate in multiple physiological activities including immune response and tissue renewal. Oxidative stress has been identified as a key factor in the breakdown of intestinal function in pigs [18]. H_2_O_2_ is a highly reactive oxygen species and serves as a signaling molecule in the regulation of a wide variety of biological processes [6–8]. H_2_O_2_ is often used in *in vitro* studies of redox-regulated processes because it is relatively stable *in vivo* compared to other reactive oxygen species.

Our results indicated that H_2_O_2_ stimulation significantly decreased cell viability and increased LDH activity in the IPEC-1 cell supernatant, demonstrating a role for H_2_O_2_ in cell injury. In agreement with our study, Zheng et al. found that incubation with H_2_O_2_ caused significant damage to nucleus pulposus cells, as indicated by an increase in apoptotic cells [19]. Similarly, H_2_O_2_ has been reported to decrease the cell viability and increase cell apoptosis in human umbilical vein endothelial cells [20]. H_2_O_2_ can also cause high death rates and induce chronic intestinal injury in mice, as demonstrated by increased apoptotic intestinal epithelial cells and damaged intestinal morphology [21]. The intestinal barrier is composed of a layer of columnar epithelium and intraepithelial tight junctions. Tight junction proteins, such as claudins, occludins, and zonula occludins, form the tight junction, work as a rate-limiting step in the paracellular pathway, and form a selectively permeable barrier [22]. We determined the distribution of tight junction claudin-1 and found that H_2_O_2_ challenge disrupted the tight junction distribution when compared to control, demonstrating a role for H_2_O_2_ in severe intestinal barrier dysfunction. Our study was in agreement with Ma et al. [23] who found H_2_O_2_-induced barrier disruption in a monolayer of Caco-2 cells. An H_2_O_2_-induced increase in renal epithelial cell paracellular permeability was also demonstrated, mediated by occludin protein, possibly by a reduction in the rate of occludin movement into the tight junction region [24]. It is known that H_2_O_2_ could cause the production of reactive oxygen species, resulting in oxidative damage of the intestinal barrier and cell apoptosis and necrosis [25, 26]. Therefore, it is possible that stimulation destroyed the IPEC-1 cell structure and then disrupted the distribution of the tight junction protein in the cell membrane.

Oxidative stress is an important driving force for an enhanced proinflammatory cytokine production in intestinal epithelial cells, causing gut mucosal inflammation. Several cytokines are now known to contribute to tissue injury and cell death, including TNF-*α*, interferon-*γ*, IL-1*β*, and IL-18 [27]. In the present study, we found that 0.5 mM H_2_O_2_ significantly increased the mRNA and protein expression of IL-6, IL-8, and TNF-*α*. Previous studies have reported that acute oxidative stress affected IL-8 and TNF-*α* expression in IPEC-J2 porcine epithelial cells [28]. It has been found that short-term H_2_O_2_ and TNF-*α* incubation induced IL-8 secretion in Caco-2 and HT-29 cells [29].

We further explored the signaling pathways involved in proinflammatory cytokine regulation. It is well known that inflammation can result in tissue injury through multiple signaling pathways. NF-*κ*B signaling is a key pathway whose activation can lead to proinflammatory cytokine production. MAPKs are a family of serine threonine kinases and are able to transduce signals from a diverse array of extracellular stimuli including oxidative stress and cytotoxic factors [30]. The extracellular regulated kinases (ERK1/2), p38 MAPK, and JNK represent the three primary MAPK signaling pathways. We found that H_2_O_2_ stimulation significantly enhanced NF-*κ*B pathway activation and increased its translocation to the nucleus. As well as canonical NF-*κ*B pathways, MAPK pathways have been reported to be involved in proinflammatory cytokine regulation [31]. We also detected MAPK activation in this study. Our results demonstrated that H_2_O_2_ stimulation activated the phosphorylation of p38 and JNK in IPEC-1 cells but had no significant effect on phosphorylation of ERK. In agreement with our results, Zhou et al. [32] reported that H_2_O_2_ stimulation activated the p38 signaling pathway in mouse intestinal epithelial cells, potentially causing apoptosis. H_2_O_2_ stimulation has been reported to activate JNK and nuclear NF-*κ*B phosphorylation in human HepG2 cells [33]. The majority of studies have demonstrated that JNK activation ultimately leads to cellular damage. Therefore, it may be possible that activation of MAPKs and NF-*κ*B signaling pathways contribute to the cell damage associated with H_2_O_2_.

To further explore the underlying mechanism of cell injury induced by H_2_O_2_, we used MAPK and NF-*κ*B inhibitors to block their signaling pathways in order to evaluate their roles in cell injury. As expected, pretreatment with the p38 inhibitor SB202190, JNK inhibitor PD98059, and NF-*κ*B inhibitor (Bay11-7082) significantly decreased IL-6, IL-8, and TNF-*α* gene expression. Similarly, other studies have demonstrated that p38 and JNK signaling pathways could activate proinflammatory cytokine release in numerous cell types [34–37]. It was found by Kim and Lee that inhibition of p38 and JNK signaling reduced the lipopolysaccharide-induced production of inflammatory mediators in keratinocytes [37]. It was also reported that inhibition of the p38 signaling pathway could suppress apoptosis and expression of proinflammatory cytokines in human chondrocytes [38]. Our results demonstrated that inhibition of MAPKs and NF-*κ*B signaling pathways could prevent the cell inflammatory response induced by H_2_O_2_ stimulation.

To further determine whether p38, JNK, and NF-*κ*B signaling pathways are important for the induction of the cell injury after H_2_O_2_ stimulation, we measured cell injury after treatment with MAPK and NF-*κ*B inhibitors. Unexpectedly, we found that inhibiting MAPK and NF-*κ*B signaling pathways did not attenuate the cell injury as indicated by decreased cell viability and increased LDH in the cell supernatant. Bay11-7082 further decreased the cell viability after H_2_O_2_ stimulation. That is because Bay11-7082 can inhibit the cell viability and promote apoptosis. These data suggested that p38, JNK, and NF-*κ*B signaling pathways were not the principle pathways mediating cell injury in IPEC-1 cells. In fact, cell death *in vivo* can trigger an inflammatory response. Damaged cells release “danger signals” that alert the host to cell death. Some of these molecules are recognized by cellular receptors that can stimulate the generation of proinflammatory mediators [39]. Dead cells can also release danger signals activating dendritic cells and promoting the generation of an immune response to antigens in and around the dying cells. For example, factor-related apoptosis suicide (FAS) is a prototypical death receptor which stimulates cells to undergo apoptosis when activated [40]. Injection of an agonistic form of an anti-FAS antibody into mice causes hepatocytes (which express Fas) to undergo apoptosis and generate a very strong inflammatory response [37]. If apoptosis is blocked, however, this inflammatory response is inhibited [41]. It is possible that the stressful stimuli (oxidative stress) firstly lead to cell damage and then induce an inflammatory response. Our results led us to speculate that proinflammatory cytokines were not the factors leading to cell injury in IPEC-1 cells after H_2_O_2_ stimulation. It is also known that dead cells can induce inflammation by releasing proinflammatory signal(s). Taking the inflammation index into consideration, it is possible that, in our study, H_2_O_2_ first induced cell death and then induced a secondary release of proinflammatory cytokines.

From our results, H_2_O_2_ caused a cell inflammatory response through the activation of MAPK and NF-*κ*B pathways in IPEC-1 cells. However, MAPK and NF-*κ*B pathways were not the specific pathways involved in the mediation of cell injury. Many other pathways such as those involved in apoptosis, necrosis, and autophagy may be involved in the cell damage induced by H_2_O_2_ [42]. There is a need for further research to determine the contribution of cell death in the regulation of inflammation and to improve our ability to distinguish these cytotoxic mechanisms from other pathways by which death-related molecules regulate inflammation. Such progress will provide us with better tools with which to clarify the role of cell death in inflammation. It will also pave the way for the development of new therapies whereby cell death can be targeted and manipulated, ultimately modulating the course of inflammation.

## 5. Conclusion

In conclusion, the present study demonstrates that H_2_O_2_ stimulation can cause IPEC-1 cell injury and alter the distribution of tight junction proteins, leading to the inflammatory response and activation of p38, JNK, and NF-*κ*B signaling pathways. Inhibiting p38, JNK, and NF-*κ*B signaling pathways prevents the cell inflammatory response but does not alleviate the cell injury. Therefore, it is suggested that H_2_O_2_ stimulation firstly leads to cell injury, followed by an inflammatory response in IPEC-1 cells.

## Figures and Tables

**Table 1:** Primer sequences used for real-time PCR.

Primer name	Primer sequence (5′-3′)	Product size (bp)	Accession numbers
*IL-6*	F: AAGGTGATGCCACCTCAGAC	151	JQ839263.1
	R: TCTGCCAGTACCTCCTTGCT		

*IL-8*	F: ACAGCAGTAACAACAACAAG	117	NM_213867.1
	R: GACCAGCACAGGAATGAG		

*TNF-α*	F: TCCAATGGCAGAGTGGGTATG	67	NM_214022.1
	R: AGCTGGTTGTCTTTCAGCTTCAC		

*β-Actin*	F: TGCGGGACATCAAGGAGAAG	194	AF017079.1
	R: AGTTGAAGGTGGTCTCGTGG		

**Table 2:** Effect of H_2_O_2_ on the mRNA expression of proinflammatory cytokines in IPEC-1 cells.

Items^1^	Control	0.5 mM H_2_O_2_	*P* value
IL-6	1.00 ± 0.05^a^	4.89 ± 0.51^b^	<0.001
IL-8	1.00 ± 0.05^a^	4.19 ± 0.14^b^	<0.001
TNF-*α*	1.00 ± 0.03^a^	6.51 ± 0.10^b^	<0.001

^ab^Means within a row differ significantly (*P* ≤ 0.05). ^1^The 2^−ΔΔCt^ method was used to analyze the relative gene expression (fold changes), which was calculated relative to the values in samples from the control group.

**Table 3:** Effect of H_2_O_2_ on the protein expression of proinflammatory cytokines in IPEC-1 cells.

Items	Control	0.5 mM H_2_O_2_	*P* value
IL-6 (pg/mL)	10.04 ± 1.14^a^	13.40 ± 1.49^b^	0.017
IL-8 (pg/mL)	15.85 ± 0.67^a^	219.31 ± 2.14^b^	<0.001
TNF-*α* (pg/mL)	7.88 ± 0.38^a^	10.99 ± 0.49^b^	<0.001

^ab^Means within a row differ significantly (*P* ≤ 0.05).

**Table 4:** Effect of Bay11-7082 on mRNA expression of proinflammatory cytokines in IPEC-1 cells stimulated with H_2_O_2_.

Items^1^	-H_2_O_2_		+H_2_O_2_		*P* value		
	Control	Bay11-7082	Control	Bay11-7082	Bay11-7082	H_2_O_2_	Interactions
IL-6	1.00 ± 0.10	0.50 ± 0.07	1.27 ± 0.02	0.78 ± 0.03	<0.001	0.002	0.898
IL-8	1.00 ± 0.03^b^	0.63 ± 0.02^a^	2.48 ± 0.15^c^	0.52 ± 0.06^a^	<0.001	<0.001	<0.001
TNF-*α*	1.00 ± 0.05^a^	0.80 ± 0.08^a^	15.75 ± 0.7^c^	3.05 ± 0.18^b^	<0.001	<0.001	<0.001

^a-c^Means within a row with different letters differ significantly (*P* ≤ 0.05). ^1^The 2^−ΔΔCt^ method was used to analyze the relative mRNA expression (fold changes), calculated relative to the values in the control group.

**Table 5:** Effect of SB202190 on the mRNA expression of proinflammatory cytokines in IPEC-1 cells stimulated with H_2_O_2_.

Items^1^	-H_2_O_2_		+H_2_O_2_		*P* value		
	Control	SB202190	Control	SB202190	SB202190	H_2_O_2_	Interactions
IL-6	1.00 ± 0.05^b^	0.29 ± 0.01^a^	5.33 ± 0.28^d^	2.31 ± 0.14^c^	<0.001	<0.001	<0.001
IL-8	1.00 ± 0.09	0.54 ± 0.01	4.61 ± 0.58	3.04 ± 0.22	0.012	<0.001	0.112
TNF-*α*	1.00 ± 0.10^a^	0.86 ± 0.14^a^	6.09 ± 0.35^c^	2.84 ± 0.21^b^	<0.001	<0.001	<0.001

^a-d^Means within a row with different letters differ significantly (*P* ≤ 0.05). ^1^The 2^−ΔΔCt^ method was used to analyze the relative mRNA expression (fold changes), calculated relative to the values in the control group.

**Table 6:** Effect of PD98059 on the mRNA expression of proinflammatory cytokines in IPEC-1 cells stimulated with H_2_O_2_.

Items^1^	-H_2_O_2_		+H_2_O_2_		*P* value		
	Control	PD98059	Control	PD98059	SB202190	H_2_O_2_	Interactions
IL-6	1.00 ± 0.11^a^	0.82 ± 0.1^a^	1.69 ± 0.11^b^	1.24 ± 0.14^a^	0.001	<0.001	<0.042
IL-8	1.00 ± 0.24^a^	0.88 ± 0.03^a^	27.90 ± 1.72^c^	22.64 ± 2.85^b^	0.024	<0.001	0.029
TNF-*α*	1.00 ± 0.10^a^	0.85 ± 0.23^a^	41.63 ± 5.69^c^	10.86 ± 1.74^b^	<0.001	<0.001	<0.001

^a-c^Means within a row with different letters differ significantly (*P* ≤ 0.05). ^1^The 2^−ΔΔCt^ method was used to analyze the relative mRNA expression (fold changes), calculated relative to the values in the control group.

**Table 7:** Effect of Bay11-7082 on protein expression of proinflammatory cytokines in IPEC-1 cells stimulated with H_2_O_2_.

Items	-H_2_O_2_		+H_2_O_2_		*P* value		
	Control	Bay11-7082	Control	Bay11-7082	Bay11-7082	H_2_O_2_	Interactions
IL-6 (pg/mL)	11.54 ± 0.82^b^	12.03 ± 1.27^b^	225.26 ± 2.02^a^	14.91 ± 2.08^b^	0.008	<0.001	0.004
IL-8 (pg/mL)	14.90 ± 0.97^d^	61.05 ± 5.89^c^	2222.20 ± 7.75^a^	119.30 ± 4.58^b^	<0.001	<0.001	<0.001
TNF-*α* (pg/mL)	5.55 ± 0.35^ab^	3.13 ± 0.32^b^	8.65 ± 1.95^a^	5.93 ± 1.26^ab^	0.047	0.025	0.917

^abcd^Means within a row differ significantly (*P* ≤ 0.05).

**Table 8:** Effect of SB202190 on the protein expression of proinflammatory cytokines in IPEC-1 cells stimulated with H_2_O_2_.

Items	-H_2_O_2_		+H_2_O_2_		*P* value		
	Control	SB202190	Control	SB202190	SB202190	H_2_O_2_	Interactions
IL-6 (pg/mL)	12.29 ± 1.55^b^	14.34 ± 1.28^b^	26.11 ± 2.27^a^	14.91 ± 1.08^b^	0.012	<0.001	0.001
IL-8 (pg/mL)	17.80 ± 3.16^c^	25.53 ± 2.39^c^	228.08 ± 7.45^a^	71.83 ± 2.72^b^	<0.001	<0.001	<0.001
TNF-*α* (pg/mL)	4.93 ± 0.58^b^	2.60 ± 0.23^b^	8.40 ± 1.62^a^	2.88 ± 0.54^b^	0.001	0.026	0.049

^ab^Means within a row differ significantly (*P* ≤ 0.05).

**Table 9:** Effect of PD98059 on the protein expression of proinflammatory cytokines in IPEC-1 cells stimulated with H_2_O_2_.

Items	-H_2_O_2_		+H_2_O_2_		*P* value		
	Control	PD98059	Control	PD98059	SB202190	H_2_O_2_	Interactions
IL-6 (pg/mL)	11.91 ± 1.04^b^	15.34 ± 1.42^b^	25.69 ± 1.60^a^	16.96 ± 0.82^b^	0.047	<0.001	<0.001
IL-8 (pg/mL)	16.35 ± 1.78^c^	18.02 ± 0.78^c^	224.54 ± 8.28^a^	76.97 ± 3.64^b^	<0.001	<0.001	<0.001
TNF-*α* (pg/mL)	5.24 ± 0.36^b^	3.40 ± 0.38^b^	8.50 ± 1.33^a^	4.51 ± 0.42^b^	<0.001	<0.001	<0.001

^abc^Means within a row differ significantly (*P* ≤ 0.05).

## Data Availability

The data used to support the findings of this study are included within the article.
